# *Chlamydia Trachomatis* and *Neisseria Gonorrhoeae* prevalence among women of reproductive age living in urogenital schistosomiasis endemic area in Ghana

**DOI:** 10.1186/1756-0500-7-349

**Published:** 2014-06-09

**Authors:** Dzidzo Yirenya-Tawiah, Ted N Annang, Kwesi A Apea-Kubi, George Lomo, David Mensah, Lorenzo Akyeh, Kwabena M Bosompem

**Affiliations:** 1 Institute for Environment and Sanitation Studies, University of Ghana, Legon, Ghana; 2 Department of Obstetrics and Gynaecology, University of Ghana Medical School, Korle-bu, Accra, Ghana; 3 Ridge Hospital, Accra, Ghana; 4 Noguchi Memorial Institute of Medical Research (NMIMR), Legon, Ghana

**Keywords:** Chlamydia, Gonorrhoeae, Urogenital schistosomiasis, Ghana

## Abstract

**Background:**

Many studies have shown an overlap in the epidemiology of sexually transmitted infections (STIs) and urogenital schistosomiasis among young women living in schistosomiasis endemic areas. Yet we found no study assessing the prevalence of STI infections in urogenital schistosomiasis endemic areas in Ghana. As part of an epidemiological study on urogenital schistosomiasis and HIV, we sought to assess the prevalence of both *Chlamydia trachomatis* (CT) and *Neisseria gonorhoeae* (NG) infections among women living in schistosomiasis endemic communities and explore the relationship between the sexually transmitted infections (STIs) and demographic characteristics, sexual behaviour and self-reported symptoms.

**Methods:**

This was a cross-sectional study in which endocervical samples were collected from 191 women aged 15–49 years from October 2005 to March 2006. Samples were examined for CT and NG using Polymerase Chain Reaction (PCR). A structured questionnaire was also used to elicit information on study participant’s gynaecological and obstetric history and symptoms for genital infection. Chi-square test and binary logistic regression were used to assess association between CT and NG and other variables such as age, sexual behaviour and self-reported symptoms.

**Results:**

The overall prevalence of CT and NG were 6.3% and 2.6% respectively.The highest prevalence rates of CT were in the 15 to 19 year group while only individuals between 15 and 39 years were positive for NG. There was no association between CT and age, contraceptive use and the other variables assessed. NG on the other hand was found to be associated with age, number of births and number of sexual partners only by chi-square test.

**Conclusions:**

Our research revealed higher prevalence of CT and NG infections when compared to previous studies conducted among higher risk groups in non-urogenital schistosomiasis areas in Ghana. We therefore recommend further studies of these STIs in urogenital schistosomiasis endemic areas in the country.

## Background

Globally, sexually transmitted infections (STIs) are of public health concern. It is estimated, that 499 million new curable STI cases occur annually [1]. Among the broad range of bacterial STIs, CT and NG are the most prevalent bacterial STIs, with sub-Saharan Africa having the highest prevalence rates of these infections [2].

In most cases, women infected with CT and NG are asymptomatic [3]. Risk factors include young age, female, number and type of sexual partners and contraceptive use [4-6]. Untreated CT and NG infections have been associated with pelvic inflammatory diseases (PID): infertility, ectopic pregnancy, chronic pelvic pain, cervical cancer, spontaneous abortion, premature delivery and low birth weight [7]. Studies suggest the possibility of women with untreated urogenital schistosomiasis also present with similar adverse outcomes [8-11]. According to Gray et al. [12], CT and NG facilitate the susceptibility of women to HIV infection.

It has also been hypothesized that *Schistosoma haematobium* infection may increase the susceptibility to HIV infection [13]. Leutscher et al. [14] also reports overlap of STIs and schistosomiasis among young adults living in urogenital schistosomiasis endemic areas. This overlap occurs at the time when urogenital schistosomiasis infection prevalence and intensity peaks and begins to decrease in young adults, while at the same time they begin to acquire sexually transmitted infections (STIs); this predisposes them to concomitant STI /schistosomiasis infection.

While urogenital schistosomiasis is endemic in Ghana, we found no studies assessing the prevalence of STI infections in these areas. According to Apea-Kubi et al. [15] most studies on CT and NG in Ghana have targeted high risk groups, hospital attendees and populations in urban settings. We therefore sought to assess the prevalence of both CT and NG among women living in rural urogenital schistosomiasis endemic communities and explore the association between the STIs and various factors that may promote infection.

## Methods

### Study design

This study was part of a larger cross-sectional epidemiological survey on urogenital schistosomiasis and HIV in the Volta Basin of Ghana involving 1,695 women. Out of these, 420 (24.8%) women aged 15–49 years, voluntarily enrolled for the study on female genital schistosomiasis (FGS) and HIV which involved screening for genital infections and HIV [16]. Out of the 420, 191 of them were randomly screened for CT and NG. The study area spanned the Afram arm of the Volta Basin to about 40 km downstream below the Akosombo dam. The study communities are all rural and located along the Volta River. Major occupations engaged in are fishing, farming and petty trading as shown by the demographic characteristic of study participants in Table 1. The overall adult (categorized as persons 15 years and above) urinary schistosomiasis prevalence rate in the study area was 46.5%. The prevalence rate of the disease in males and females is 56.5% and 36.9% respectively [16].

**Table 1:** Demographic characteristics of women enrolled in the epidemiological survey

**Age group (years)**	**Number (%)**
**Age (years)**	
15-24	35 (18.2)
25-34	72 (37.7)
> 34	84 (44.0)
**Marital status**	
Single	38(19.9)
Married	142 (74.3)
Divorced	6(3.1)
Widowed	5 (2.6)
**Education level**	
Illiterate	68 (35.6)
Primary	83 (43.5)
JHS	32 (16.8)
SHS	8 (4.1)
Tertiary	0 (0.0)
**Occupation**	
Schooling	9 (4.7)
Fishing	4 (2.1)
Farming	68 (35.6)
Trading	93 (48.7)
Unemployed	6 (3.1)
Others	11 (5.7)
**Length of stay in village (years)**	
Less than 1	4 (2.1)
Between 1 and 2	4 (2.1)
Between 2 and 5	18 (9.4)
Between 5 and 10	51 (26.7)
More than 10	114 (59.7)

JHS-Junior High Secondary.

SHS-Senior High Seconday.

The eligibility criteria and recruitment procedure used in this study has already been described elsewhere [17]. Briefly, study participants who voluntarily agreed to participate in the study were recruited from the larger study after informed verbal consent was provided. All participants prior to screening were administered a structured questionnaire that focused on their obstetric and gynaecological history and self-reported gynaecological symptoms observed at the time of the study. This questionnaire was in English but was administered in private by Research Fellows and trained Research Assistants. Questionnaires were administered in the local languages spoken by study participants; Ewe, Dangme and Twi.

After responding to the questionnaires, all participants were physically examined by a gynaecologist, and we excluded pregnant women , virgins, those who have had surgery, those who had the presence of any health condition (e.g. diabetes, hypertension etc.) and women menstruating at the time of the study. The inclusion criteria were:

1. Age between 15 and 49 years

2. Sexually active

3. Living in study area for over a year

4. Willingness to participate in study

Additionally, the gynaecologist physically examined the external and internal genitalia for any abnormality and these were recorded. Endocervical specimens were collected and placed immediately into 2SP transport medium (2 M sucrose and 0.02 M phosphate) (Roche Diagnostics, Indianapolis USA) and transported on ice at 4°C to Noguchi Memorial Institute for Medical Research (NMIMR) to be tested for C*hlamydia trachomatis and Neisseria gonorrhoeae* detection.

CT and NG were diagnosed using the Amplicor *C. trachomatis/N. gonorrheae* assay (Roche Diagnostic Corporation, Indianapolis, IN, USA). All tests were done according to the manufacturer’s description/instruction on the package Van der pol *et al*. [18].

Ethical approval for the study was granted by the Noguchi Memorial Institute for Medical Research, Institutional Review Board. Consent was also granted by District Health Management teams, Community leaders, Community members and study participants. Participants who tested positive for both CT and NG were treated by the gynecologist according to stipulated national guidelines.

### Data analysis

All data were double entered in Microsoft excel (Microsoft Corporation, Redmond, WA, USA) to ensure accuracy and later transferred into SPSS version 18 for analysis. First, descriptive statistics were done which was followed by using the Chi-square test and logistic regression to determine association between CT and NG infections, the dependent variables and independent variables such as age, sexual behaviour and self-reported symptoms. All analyses performed excluded missing data and were conducted at 95% confidence intervals (CI).

## Results

Out of the 191 women screened for CT and NG in this study, 12(6.3%) and 5(2.6%) were positive for CT and NG respectively, and 2 (1.0%) co-infected with both CT and NG. The majority of study participants who were diagnosed with CT were within the age group 15–24 years (Table 2). The results from Table 2 also show that, there was no association between CT and all the factors assessed (age, number of births, sexual behaviour).

**Table 2:** Association between CT and selected characteristics of study subjects

	**CT infection status**		**Measure of association**		
**Characteristics**	**Number positive (%)**	**Number negative (%)**	**Chi-square (P value)**	**OR(CI)**	**P value**
**Age group (N = 186)**					
15-24	4 (11.4)	31 (88.6)	2.6 (0.3)	3.4 (0.7-16.5)	0.1
25-34	5 (6.9)	67 (93.1)		2.0 (0.4-8.7)	0.3
* ≥ 35	3 (3.6)	81 (96.4)			
Number of births (N = 190)					
0	3 (13.6)	19 (86.4)		3.4 (0.7-16.6)	0.1
1-3	5 (5.9)	73 (93.6)	2.6 (0.3)	1.4 (0.4-5.8_)	0.6
* ≥ 4	4 (4.4)	87 (95.6)			
Number of sexual partners in past one year (N = 191)					
0	0 (0.0)	10 (100.0)	1.4 (0.4)	1.0	1.0
1	11 (6.3)	163 (93.7)		1.0	1.0
2	1(16.7)	5 (83.3)		3.2	1.0
* ≥ 3	0 (0.0)	1 (100)			
**Age at first intercourse (N = 189)**					
9-15	4 (8.2)	45 (91.8)	1.0 (0.8)	1.4	0.9
16-22	8 (6.1)	123 (93.9)		1.1	0.9
≥23	0(0.0)	11 (100.0)			
**Frequency of sexual intercourse (N = 187)**					
Every day	0 (0.0)	2 (100.0)		0.0 (0.0)	0.9
Once a week	9 (6.8)	123 (93.2)	0.9 (0.9)	0.0 (0.0)	0.9
Twice in a month	0 (0.0)	7 (100.0)		1.05 (0.3-4.1)	0.9
≤ once a month	3 (6.5)	43 (93.5)			
**Contraceptive use (N = 191)**					
No contraceptive use	11 (7.4)	137 (92.6)		2.8 (0.4-23.0)	0.3
Hormonal contraceptive use	0 (0.0)	6 (100.0)	0.8 (0.6)	0.0 (0.0)	0.9
*Non hormonal contraceptive use	1 (2.7)	36 (97.3)			

*Variables used as reference category in regression model.

OR- Odds Ratio; CI- Confidence Interval.

where p-value is 0.9 and 1, no CI was computed by model.

Table 3 shows the relationship between NG infection and factors studied. All the subjects infected with NG were between the ages of 15 to 34 years. In analyzing for any association between NG and the various factors by chi-square test, the study showed a significant difference between NG and age (p = 0.002), NG and number of births a woman has undergone (p = 0.04) and NG and number of sexual partners a woman had in the past year (p = 0.00). We also found only 1.0% of women who had never given birth and 1.6% of women who had given birth 1 to 3 times to be infected with NG. The rest of the women who had given birth 4 times and more were NG negative (Table 3). The majority of participants (174) had had one sexual partner in the preceding year to the study. Regarding having multiples sexual partners, only 6 participants reported having 2 sexual partners and 1 participant reported having 5 sexual partners in the year preceding this study. Ten of them indicated they had had no sexual partners in the past year (Table 3). Among subjects found to be positive for NG, 1.6% of them had no sexual partner and 1.0% had a single sexual partner. There was no association found between number of sexual partners and NG infection and contraceptive use and NG both by chi-square and logistic regression.

**Table 3:** Association between NG and selected characteristics of study subjects

**Characteristics**	**No. positive (%)**	**No. negative (%)**	**Chi-square P value**	**OR (CI)**	**P value**
Age group (N = 186)					
15-24	3 (8.6)	32 (91.4)	7.0 (0.03))	1.5 (0.0)	0.9
25-34	2 (2.8)	70 (97.2)		4.6 (0.0)	0.9
* ≥ 35	0 (0.0)	84 (100.0)			
Number of births (N = 190)					
0	3 (13.6)	19 (86.4)	12.9	2.5 (0.0)	1
1-3	2 (2.6)	76 (97.4)	(0.002)	4.2 (0.0)	1
* ≥ 4	0 (0.000)	91 (100.0)			
Number of sexual partners in past one year (N = 191)					
0	0 (0.0)	10 (100.0)	23	1.0 (0.0)	1.0
1	3 (1.7)	171 (98.3)	(0.00)	2.8(0.0)	1.0
2	2 (33.3)	4 (66.7)		8.0(0.0)	1.0
≥3	0 (0.0)	1 (100.0)			
Age at first intercourse (N = 189)					
9-15	1 (2.0)	48 (89.0)		3.3	1.1
16-22	4 (3.1)	127 (96.9)	0.6 (0.9)	5.0	1.1
≥23	0(0.0)	11 (100.0)			
Frequency of sexual intercourse (N = 187)					
Every day	0 (0.0)	2 (100.0)	2.2 (0.))	1.0	1.0
Once a week	5 (3.8)	127 (96.2)		6.3	0.9
Twice in a month	0 (0.0)	7 (100.0)		1.0	1.0
≤ once a month	0 (0.0)	46 (100.0)			
Contraceptive use (N = 191)					
No contraceptive use	4 (2.1)	144 (75.4)	0.2 (1.0)	1.0	1
Hormonal contraceptive use	0 (0.0)	6 (100.0)		0.0	0.9
*Non hormonal contraceptive use	1 (0.5)	36 (18.8)			

*Variables used as reference category in regression model.

OR- Odds Ratio; CI- Confidence Interval.

where p-value is 0.9 and 1, no CI was computed by model.

Tables 4 and 5 also show the distribution of self-reported symptoms by CT and NG infection status respectively. The study revealed that the majority of participants with self-reported symptoms were negative for CT and NG respectively. There was also no association found between the STIs and any of the reported symptoms both by chi-square and logistic regression.

**Table 4:** Association between CT infection and self- reported symptom

**Symptoms**	**CT status**		**Measure of association**		
	***No. positive (%)***	***No. negative (%)***	***Chi-square***	***OR (CI)***	***p value (CI)***
Vaginal itch (N = 120)					
Yes	6 (7.3)	76 (92.7)	0.1	1.1 (0.1-2.0)	0.9
*No	3 (7.9)	35 (92.1)			
Lower abdominal pain (N = 191)					
Yes	7 (6.6)	99 (93.4)	0.4	0.9 (0.3-5.0)	0.8
*No	5 (5.9)	80 (94.1)			
Dyspareunia (N = 191)					
Yes	4 (5.3)	71 (94.7)	0.2	1.3 (0.4-4.5)	0.6
*No	8 (6.9)	108 (93.1)			
Vaginal discharge (N = 191)					
Yes	9 (7.7)	108 (92.3)	1.0	0.5 (0.1-2.0)	0.3
*No	3 (4.1)	71 (95.9)			

*Variables used as reference category in regression model.

OR- Odds Ratio; CI- Confidence Interval.

**Table 5:** Association between NG infection and self- reported symptom

**Symptoms**	**NG status**		**Measure of association**		
	***No. positive (%)***	***No. negative (%)***	***Chi-square***	***OR (CI)***	***p value***
Vaginal itch (N = 120)					
Yes	3 (3.7)	79 (92.3)	0.08 (1.0)	0.7 (0.07-7.1)	0.7
*No	1 (2.6)	37 (94.4)			
Lower abdominal pain (N = 191)					
Yes	2 (1.9)	104 (98.1)	0.5 (0.7)	1.9 (0.3-11.7)	0.4
*No	3 (3.5)	82 (96.5)			
Dyspareunia (N = 191)					
Yes	4 (5.3)	72 (94.7)	3.5 (0.08)	2.8 (0.3-25)	0.4
*No	1 (0.9)	115 (99.1)			
Vaginal discharge (N = 191)					
Yes	4 (3.4)	113 (96.6)	0.8 (0.7)	0.4 (0.004-3.5)	0.4
*No	1 (1.4)	73 (98.6)			

*Variables used as reference category in regression model.

OR- Odds Ratio; CI- Confidence Interval.

## Discussion and conclusions

This study reports on CT and NG prevalence among women of reproductive age living in rural urogenital schistosomiasis communities in the Volta Basin of Ghana. The prevalence levels found were 6.3% and 2.3% for CT and NG respectively. Earlier prevalence studies conducted in Ghana among high risk populations (gynaecologic clinic attendees, women recruited from drinking bars, night clubs, female hostels and work places of apprentice seamstresses and hairdressers) reported CT prevalence rates ranging from 1.5% to 4.8% and NG prevalence rates ranging from 0 to 3.1% [4,15,19,20]. Another study conducted in a health facility in Zambia found prevalence rate of CT and NG among HIV infected women to be 1% and 1.4% respectively [21]. On the other hand, the CT prevalence rate determined by our study is consistent with findings from a similar study by Leutscher et al. [14] in a urogenital schistosomiasis community in Madagascar where they found CT prevalence rate of 9.8% among their study participants.

The higher prevalence of CT reported by this study when compared to others conducted in Ghana in non-urogenital schistosomiasis endemic areas reported may be explained by the fact, that schistosomiasis infection which usually occurs in childhood among people living in endemic areas may compromise the reproductive tract by causing ulceration and thinning of the epithelium before an individual reaches the sexual active age. This allows for easy penetration and implantation of any STI that may be contracted when an already positive schistosomiasis case becomes sexually active as a young adult [22]. In a community based study in Northern Tanzania, Poggensee et al*.*[23] found high prevalence of FGS in all age groups and the high levels of pathologic cervical alterations such as swollen and disrupted epithelium, supporting the hypothesis that FGS was a risk factor for the transmission of STI infections.

Our findings did not reveal any association between self-reported symptoms and STIs studied. Nevertheless, we observed only a few, less than 10.0%, of the STI positive cases to report the presence of some symptom(s). As shown by Romoren et al. [2], many CT and NG infected individuals are asymptomatic. A meta-analysis of several studies by Wasserheit [24] demonstrated, that only 28% of women with CT and NG infection reported symptom of vaginal discharge. She also found, that 92% of cases with vaginal discharge to be neither infected with CT nor NG. Similar observations were also made by this study. As such this study supports earlier suggestions that a syndromic approach should not be used as a screening tool for CT and NG [25-28].

Our study was limited in a number of ways. Firstly, the fact that participation was on a voluntary basis created selection bias and therefore our sample participants may not be representative of the population in the study area. Also, our study participants presented (with) similar characteristics and this may account for the weak relationship observed between the infections and the factors studied. For instance the majority of the participants were married and were not expected to have multiple partners, hence it was not surprising that there was no association between number of sexual partners and CT and NG. Likewise, contraceptive use in most African rural settings, particularly in marital relationships is low. It was therefore expected that contraceptive use would be low and this also accounted for the fact that no association was observed between STIs and contraceptive used. Secondly, because of the small sample size, study results may not be robust and therefore should be interpreted with caution. Thirdly, we did not consider antibiotic usage among participants which may have also impacted on STI prevalence rates observed.

This study, nevertheless, serves as a baseline study for future studies on STIs in schistosomiasis endemic areas. There is a need for community screening and treatment, and health educational campaigns on STI infections in urogenital schistosomiasis endemic communities in the country.

## Competing interests

The authors declare that they have no competing interests.

## Authors’ contributions

Contribution of the authors is as follows; DYT, KAAK and KMB designed the study and wrote the protocol. DYT, TA, KAAK, GL and LA collected data. KAAK and GL collected gynaecological samples, and LA conducted bacteriological assessment. Authors DYT and DM, worked on the data analysis and DYT and KMB drafted the manuscript. All authors read and approved the final manuscript.

## References

[B1] WHO Sexually transmitted infections (STIs): updated fact sheet 2013 http://www.who.int/mediacentre/factsheets/fs110/en/index.html

[B2] Romoren M Sundby J Velauthapillai M Rahman M Klouman E Hjortdahl P Chlamydia and gonorrhoea in pregnant Batswana women: time to discard the syndromic approach? BMC Infect Dis 2007 7 27 10.1186/1471-2334-7-27 17437632 PMC1955709

[B3] Turner CF Rogers SM Miller HG Miller WC Gribble JN Chromy JR Leone PA Cooley PC Quinn TC Zenilman JM Untreated gonococcal and chlamydial infection in a probability sample of adults JAMA 2002 287 6 726 733 10.1001/jama.287.6.726 11851539

[B4] Opoku BK Sarkodie Y Prevalence of genital Chlamydia and gonococcal infections in at risk women in the Kumasi metropolis Ghana Ghana Med J 2010 44 1 21 24 21326987 10.4314/gmj.v44i1.68852 PMC2956315

[B5] Einwalter LA Ritchie JM Ault KA Smithem EM Gonorrhea and Chlamydia infection among women visiting family planning clinics: racial variation in prevalence and predictors Perspect Sex Reprod Health 2005 37 3 135 140 10.1363/3713505 16150661

[B6] Centers for Disease Control and Prevention (CDC) Sexually Transmitted Disease Surveillance 2001 Atlanta: CDC

[B7] Chacko MR Wiemann CM Smith PB Chlamydia and gonorrhea screening in asymptomatic young women J Pediatr Adolesc Gynecol 2004 17 3 169 178 10.1016/j.jpag.2004.03.041 15125902

[B8] Nour NM Schistosomiasis: health effects on women Rev Obstet Gynecol 2010 3 1 28 32 20508780 PMC2876318

[B9] Swai B Poggensee G Mtweve S Krantz I Female genital schistosomiasis as an evidence of a neglected cause for reproductive ill-health: a retrospective histopathological study from Tanzania BMC Infect Dis 2006 6 134 doi:10.1186/1471-2334-6-134 10.1186/1471-2334-6-134 16928276 PMC1564144

[B10] Qunhua L Jiawen Z Bozhao L Zhilan P Huijie Z Shaoying W Delun M Hsu LN Investigation of association between female genital tract diseases and Schistosomiasis japonica infection Acta Trop 2000 77 2 179 183 10.1016/S0001-706X(00)00129-7 11080508

[B11] Poggensee G Feldmeier H Female genital schistosomiasis: facts and hypotheses Acta Trop 2001 79 3 193 210 10.1016/S0001-706X(01)00086-9 11412803

[B12] Gray RH Wawer MJ Reassessing the hypothesis on STI control for HIV prevention Lancet 2008 371 2064 2206 10.1016/S0140-6736(08)60896-X 18572064

[B13] Mbabazi PS Andan O Fitzgerald DW Chitsulo L Engels D Downs JA Examining the relationship between urogenital schistosomiasis and HIV infection PLoS Negl Trop Dis 2011 12 e1396 22163056 10.1371/journal.pntd.0001396 PMC3232194

[B14] Leutscher PD Ramarokoto CE Hoffmann S Jensen JS Ramaniraka V Randrianasolo B Raharisolo C Migliani R Christensen N Coexistence of urogenital schistosomiasis and sexually transmitted infection in women and men living in an area where Schistosoma *haematobium* is endemic Clin Infect Dis 2008 47 6 775 782 10.1086/591127 18680415

[B15] Apea-Kubi KA Shinya Y Sakyi B Kisimoto T Ofori- Adjei D Hagiwara T *Neisseria gonorrhoea*e, chlamydial trachomatis and treponema pallidum infection in antenatal and gynaecological patients at Korle-Bu Teaching Hospital Ghana Jpn J Infect Dis 2004 57 253 25616 15623949

[B16] Yirenya-Tawiah DR Annang T Otchere J Bentum D Edoh D Amoah C Bosompem K Urinary schistosomiasis among adults in the Volta Basin of Ghana: prevalence, knowledge and practice J Trop Med Parasitol 2011 34 1 16

[B17] Yirenya-Tawiah DR Amoah CM Apea-Kubi KA Dade M Lomo G Mensah D Akyeh L Bosompem KM Female genital schistosomiasis, genital tract infections and HIV co-infection in the Volta basin of Ghana Intern J Trop Dis Health 2013 3 2 94 103

[B18] Van der Pol B Quinn TC Gaydos CA Crotchfelt K Schachter J Moncada J Jungkind D Martin DH Turner B Peyton C Jones RB Multicenter Evaluation of the amplicor and automated COBAS AMPLICOR CT/NG Tests for Detection of *Chlamydia trachomatis* J Clin Microbiol 2000 38 3 1105 1112 10699004 10.1128/jcm.38.3.1105-1112.2000 PMC86350

[B19] Pepin J Deslandes S Khonde N Kintin DF Diakité S Sylla M Méda H Sobéla F Asamoah-Adu C Agyarko-Poku T Frost E Low prevalence of cervical infections in women with vaginal discharge in West Africa: implications for syndromic management Sex Transm Infect 2004 80 230 235 10.1136/sti.2003.007534 15170011 PMC1744831

[B20] Bentsi C Klufio CA Perine PL Bell TA Cles LD Koester CM Wang SP Genital infections with *Chlamydia trachomatis and Neisseria gonorrhoeae* in Ghanaian women Genitourin Med 1985 61 1 48 50 3936773 10.1136/sti.61.1.48 PMC1011755

[B21] Alcaide ML Jones DL Chitalu N Stephen W Chlamydia and gonorrhea infections in HIV-positive women in urban Lusaka, Zambia J Glob Infect Dis 2012 4 3 10.4103/0974-777X.100566 PMC3459430 23055644

[B22] Poggensee G Feldmeier H Female genital schistosomiasis: facts and hypothesis Acta Tropical 2001 79 3 193 210 10.1016/S0001-706X(01)00086-9 11412803

[B23] Poggensee G Kiwelu I Weger V Goppner D Diedrich T Krantz I Feldmeier H Female genital schistosomiasis of the lower genital tract: prevalence and disease-associated morbidity in northern Tanzania J Infect Dis 2000 181 3 1210 1213 10.1086/315345 10720558

[B24] Wasserheit J The significance and scope of reproductive tract infections among Third World women Suppl Int J Gynecol Obstet 1989 3 145 168 2686703 10.1016/0020-7292(89)90115-x

[B25] Scholes D Stergachis A Heidrich FE Andrilla H Holmes KK Stamm WE Prevention of pelvic inflammatory disease by screening for cervical chlamydial infection N Engl J Med 1996 334 21 1362 1366 10.1056/NEJM199605233342103 8614421

[B26] Pettiforn A Walsh J Wilkins V Raghunathan P How effective is syndromic management of STDs?: a review of current studies Sex Transm Dis 2000 27 371 385 10.1097/00007435-200008000-00002 10949428

[B27] Sloan NL Winikoff B Haberland N Coggins C Elias C Screening and syndromic approaches to identify gonorrhea and chlamydial infection among women Stud Fam Plann 2000 31 55 68 10.1111/j.1728-4465.2000.00055.x 10765538

[B28] Dallabetta GA Gerbase AC Holmes KK Problems, solutions, and challenges in syndromic management of sexually transmitted diseases Sex Transm Infect 1998 74 Suppl 1 S1 S11 10023346

